# Rocaglamide overcomes tumor necrosis factor-related apoptosis-inducing ligand resistance in hepatocellular carcinoma cells by attenuating the inhibition of caspase-8 through cellular FLICE-like-inhibitory protein downregulation

**DOI:** 10.3892/mmr.2014.2718

**Published:** 2014-10-21

**Authors:** ZHOU LUAN, YING HE, FAN HE, ZHISHUI CHEN

**Affiliations:** 1 Institute of Organ Transplantation, Tongji Hospital, Tongji Medical College, Huazhong University of Science and Technology, Wuhan, Hubei 430030, P.R. China; 2 Department of Ophthalmology, The Central Hospital of Wuhan, Wuhan, Hubei 430014, P.R. China

**Keywords:** rocaglamide, tumor necrosis factor-related apoptosis-inducing ligand, hepatocellular carcinoma, cellular FLICE-like inhibitory protein, caspase-8

## Abstract

The enhancement of apoptosis is a therapeutic strategy used in the treatment of cancer. Tumor necrosis factor-related apoptosis-inducing ligand (TRAIL) is a promising antitumor agent. However, hepatocellular carcinoma (HCC) cells exhibit marked resistance to the induction of cell death by TRAIL. The present study investigated whether rocaglamide, a naturally occurring product isolated from the genus *Aglaia*, is able to sensitize resistant HCC cells to TRAIL-mediated apoptosis. Two HCC cell lines, HepG2 and Huh-7, were treated with rocaglamide and/or TRAIL and the induction of apoptosis and effects on the TRAIL signaling pathway were investigated. The *in vivo* efficacy of rocaglamide was determined in TRAIL-resistant Huh-7-derived tumor xenografts. Rocaglamide significantly sensitized the TRAIL-resistant HCC cells to apoptosis by TRAIL, which resulted from the rocaglamide-mediated downregulation of cellular FLICE-like inhibitory protein and subsequent caspase-8 activation. Furthermore, rocaglamide markedly inhibited tumor growth from Huh-7 cells propagated in severe combined immunodeficient mice, suggesting that chemosentization also occurred *in vivo*. These data suggest that rocaglamide acted synergistically with TRAIL against the TRAIL-resistant HCC cells. Thus, it is concluded that rocaglamide as an adjuvant to TRAIL-based therapy may present a promising therapeutic approach for the treatment of HCC.

## Introduction

Hepatocellular carcinoma (HCC) is one of the most common and life-threatening types of human malignant tumor (1). The inadequate effects of conventional chemotherapy and resistance to drugs present a challenge to HCC treatment. In particular, advanced HCC cells respond poorly to the induction of apoptosis by chemotherapeutic agents due to reprogramming of cellular apoptotic machinery (2). Thus, there is a requirement to examine potential targets of the cellular apoptotic machinery to develop novel and potent therapeutic drugs for the treatment of HCC.

Tumor necrosis factor-related apoptosis-induced ligand (TRAIL), a type II transmembrane protein from the tumor necrosis factor family, triggers the extrinsic pathway of apoptosis by binding to the cell surface receptors death receptor (DR) 4 and 5 (3). These receptors then form homomeric and heteromeric complexes and stimulate the recruitment of Fas-associated death domain (FADD) and caspase-8, which self-activate and initiate downstream caspase cleavage events (4). TRAIL is promising as a novel therapeutic agent due to its high potential for the selective induction of apoptosis (5). However, several studies have indicated that HCC cells are relatively refractory to TRAIL and that TRAIL alone is unable to induce apoptotic cell death in these cells (6). A number of factors may be responsible for the resistance of HCC cells to TRAIL. TRAIL-induced apoptosis is mediated by caspase-8 (7) and the resistance of HCC cells to TRAIL is correlated with a downregulation in the activity of caspase-8, which disturbs apoptotic signals in the cancer cells (8). Notably, the activity of caspase-8 is inhibited by its inhibitory protein, the cellular FLICE-like inhibitory protein (c-FLIP) in chemoresistant cells (9). c-FLIP is expressed at higher levels in TRAIL-resistant HCC cells compared with TRAIL-sensitive cells (10). Furthermore, the downregulation of c-FLIP renders highly resistant HCC cells sensitive to TRAIL treatment (11). Therefore, novel therapeutic strategies to eliminate the c-FLIP-mediated chemoresistance of HCC cells are required, and the identification of novel therapeutic compounds with anti-HCC activity, particularly those derived from naturally occurring materials, is necessary.

Rocaglamide is isolated from the genus *Aglaia* (family Meliaceae) (12). A number of species from this genus are used in traditional medicine to treat coughs, injuries, asthma and inflammatory skin diseases. Rocaglamide has also been observed to possess anticancer properties in leukemia (13,14). However, the detailed mechanisms underlying the anticancer activities of rocaglamide in solid tumors remain to be elucidated.

The aim of the present study was to investigate whether rocaglamide sensitized resistant HCC cells to TRAIL-induced death, by regulation of caspase-8/c-FLIP *in vitro*. Furthermore, the efficacy of rocaglamide in TRAIL-resistant Huh-7-derived tumor xenografts was determined.

## Materials and methods

### Cell culture and reagents

HepG2 and Huh-7 cells were obtained from the Shanghai Cell Collection (Shanghai, China) and cultured in Dulbecco’s modified Eagle’s medium (DMEM; Gibco-BRL, Carlsbad, CA, USA) supplemented with 10% fetal bovine serum (FBS; Gibco-BRL), 2 mM glutamine, 100 U/ml penicillin and 100 μg/ml streptomycin. The cells were cultured at 37°C in 5% CO_2_. Rocaglamide (>98% pure) was procured from Enzo Life Sciences (Lörrach, Germany). TRAIL was purchased from PeproTech, Inc. (Rocky Hill, NJ, USA) and all chemicals were purchased from Sigma (St. Louis, MO, USA), unless indicated otherwise.

### Treatment of cells with rocaglamide and/or TRAIL

For the investigation of time-dependence, the cells (70% confluent) were treated with rocaglamide (100 nM) and/or TRAIL (100 ng/ml) for different time periods (0–24 h). The cells were then harvested for cell viability analysis. For the investigation of dose-dependence, the cells (70% confluent) were pretreated with rocaglamide (0–100 nM) for 12 h in DMEM, supplemented with 10% FBS, 2 mM glutamine, 100 U/ml penicillin and 100 μg/ml streptomycin, and were then treated with TRAIL (0–100 ng/ml) for an additional 12 h. To investigate treatment with a combination of rocaglamide and TRAIL, the cells were pretreated with either rocaglamide or dimethyl sulfoxide (DMSO) for 12 h, followed by 12 h incubation in the presence of TRAIL. Subsequently, 24 h after rocaglamide or DMSO treatment, the cells were harvested and the cell lysates were prepared, according to previous methods (15) and stored at -80°C for later use.

### Cell viability assay

HepG2 and Huh-7 cells (1×10^4^/well) were seeded in 96-well plates in complete culture medium and incubated for 24 h. The cells were then exposed to 100 nM rocaglamide and/or 100 ng/ml TRAIL for 24 h. The control cells were treated with DMSO at a concentration equal to that used for the drug-treated cells. The complete culture medium was then removed and MTT (200 μl, 0.5 mg/ml in 10% FBS-containing DMEM) was added to each well and the plate was incubated for 2 h at 37°C in a humidified incubator. The solution was then removed from the wells and 200 μl DMSO was added to each well prior to agitation. The absorbance at 570 nm was read using a microplate reader (Bio-Tek ELx800; BioTek Instruments Inc., Winooski, VT, USA). The value for the vehicle-treated cells was considered to indicate 100% viability. Furthermore, a crystal violet assay was carried out. Briefly, the cells (1.0×10^5^/ml) were seeded in a 12-well plate for 12 h, and treated with TRAIL (0–100 ng/ml) and/or RocA(1–100 nM) for 12 h. The treated cells were washed with phosphate-buffered saline (PBS), fixed with 4% paraformaldehyde for 15 min, and stained using crystal violet (Sigma; cat no. C3886) for a further 30 min.

### Western blot analysis

Western blot analysis was performed, as described previously (7) using the following: mouse anti-c-FLIP monoclonal antibody (NF6; cat.no ALX-804-428; Alexis Biochemicals, San Diego, CA, USA), mouse anti-caspase-8 monoclonal antibody (cat.no 9746; Cell Signaling Technology, Inc. Danvers, MA, USA), mouse anti-DR4 monoclonal antibody (cat.no ab13890; Abcam, Cambridge, UK), rabbit anti-DR5 polyclonal antibody (cat.no ab47179; Abcam), Pro-Apoptosis Bcl-2 Family Antibody Sampler kit (cat.no 9942; Cell Signaling Technology, Inc.) and Apoptosis Western Blot Cocktail (cat.no ab136812; Abcam). An enhanced chemiluminescence detection reagent (SuperSignal West Pico) was used for detection (Pierce Biotechnologies, Rockford, IL, USA) and α-tubulin and β-actin were used as loading controls. All western blots were representative of at least three independent experiments.

### Transfection of c-FLIP

Small interfering (si)RNA control or siRNA FLIP (Santa Cruz Biotechnology) of high purity were delivered into the HepG2 cells in the 12-well plate using Lipofectamine 2000 (Invitrogen Life Technologies, Carlsbad, CA, USA) according to the manufacturer’s instructions. Briefly, 2 μl Lipofectamine 2000 was added to 50 nmol/l siRNA in a final volume of 100 μl culture medium and the solution was added to the washed cells, which were then incubated at 37°C for 4 h. The transfection mixture was then removed and the cells were incubated with fresh complete medium for an additional 36 h. Finally, the cells were exposed to TRAIL or DMSO for 12 h for subsequent experiments.

### Detection of apoptosis and necrosis using flow cytometry

The HepG2 and Huh-7 cells were seeded and cultured in each well of a 12-well plate for 24 h and then pretreated with either rocaglamide or DMSO for 12 h, followed by 12 h incubation in the presence of TRAIL. An annexin V/propidium iodide (PI) apoptosis detection kit (eBioscience, Inc., San Diego, CA, USA) was used for the detection of apoptotic and necrotic cells. The cells (5×10^5^) were harvested and resuspended in 400 μl 1× binding buffer with 4 μl FITC annexin V and 4 μl PI. Flow cytometric analysis was then performed using a Becton-Dickinson FACSCalibur™ system; BD Biosciences, Franklin Lakes, NJ, USA) and FlowJo 7.6.3 software (Tree Star, Inc., Ashland, OR, USA).

### Tumorigenicity studies in severe combined immunodeficient (SCID) mice

The Huh-7 cells (3×10^6^), suspended in 100 μl mix (equal volumes of DMEM and Matrigel), were implanted subcutaneously into the right flank of 10 female SCID mice (6-week-old) and then randomly divided into two equal groups, one of which received an intraperitoneal injection of rocaglamide (2.5 mg/kg in 80 μl olive oil; n=5) and the other, used as a vehicle control, received olive oil alone (n=5). These treatments were performed once daily for 32 days and the tumor volumes and body weights of the animals were measured twice a week. The tumor volumes (mm^3^) were calculated using the following formula: Tumor volume = LS^2^ / 2, where L is the longest diameter and S is the shortest. At the end of the experiments, the mice were sacrificed and tumor samples were harvested, fixed in formalin and embedded in paraffin as tissue sections for immunohistochemical analysis. The animal experiments were performed in accordance with the relevant institutional and national regulations and the research procedures were approved by Tongji Medical College (Wuhan, China). The SCID mice were purchased from Beijing HFK Bioscience, Co., Ltd. (Beijing, China).

### Immunohistochemistry and terminal deoxynucleotidyltransferase-mediated dUTP nick end labeling (TUNEL) staining

Immunohistochemical staining was performed using either 1:1 hematoxylin and 0.5% eosin (H&E) or an anti-cleaved caspase-3 antibody (Cell Signaling Technology, Inc.), as previously described (16). Apoptosis was assessed by TUNEL (*In situ* Cell Death Detection kit; Roche Diagnostics, Basel, Switzerland) according to the manufacturer’s instructions. The primary antibody was omitted in the first step to establish a negative control, which yielded negative results. The percentage of positive cells was independently determined by two examiners. At least five random fields of each section were visualized (magnification ×400) and analyzed using a Nikon OPTIPHOT 150 microscope (Nikon, Tokyo, Japan) connected to a SPOT Insight charge-coupled device camera (Diagnostic Instruments, Inc., Sterling Heights, MI, USA). The values are expressed as the mean ± standard deviation (SD).

### Statistical analysis

Where indicated, the data are expressed as the mean ± SD from at least three independent experiments. Statistical analysis of the data was conducted using Student’s t-test. P<0.01 was considered to indicate a statistically significant difference.

## Results

### Rocaglamide enhances TRAIL-induced apoptosis in resistant HCC cells

**Figure 1:**
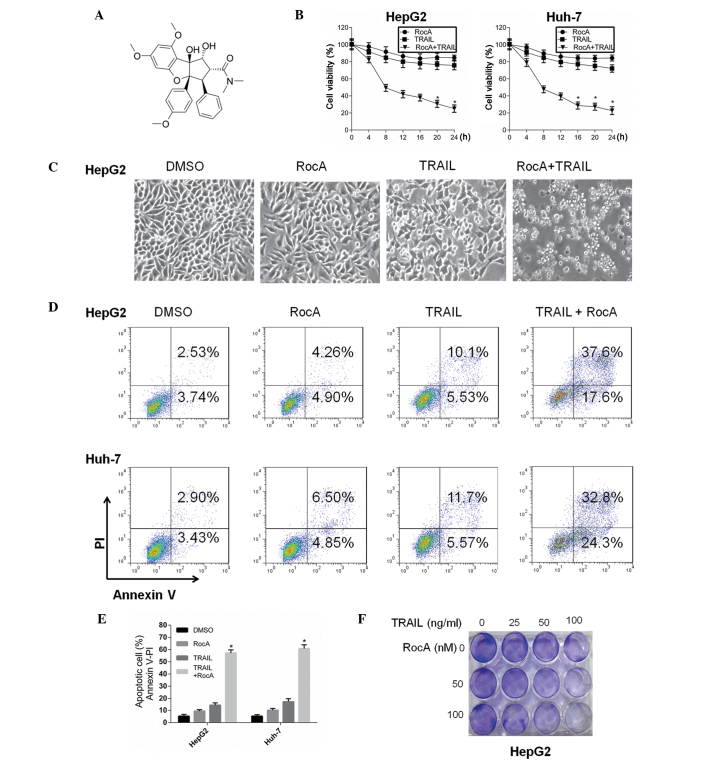
RocA sensitizes HCC cells to TRAIL-mediated apoptosis. (A) Chemical structure of RocA. (B) HepG2 and Huh-7 cells were treated with RocA (100 nM) and/or TRAIL (100 ng/ml) for different time periods, as indicated. Cell viability was measured using an MTT assay. (C) Representative images of the cells were captured using phase-contrast microscopy (magnification, ×400). (D and E) HepG2 and Huh-7 cells were pretreated with RocA (100 nM) for 12 h and then exposed to TRAIL (100 ng/ml) for 12 h. Cell apoptosis was determined using flow cytometry with annexin/PI double staining. (F) Cells were washed with PBS and fixed with 4% paraformaldehyde and were stained using crystal violet. Data are expressed as the mean ± standard deviation. ^*^P<0.01, TRAIL+RocA group vs TRAIL group. Data are representative of three experiments. RocA, rocaglamide; HCC, hepatocellular carcinoma; TRAIL, tumor necrosis factor-related apoptosis-inducing ligand; DMSO, dimethyl sulfoxide; PI, propidium iodide.

To investigate whether rocaglamide enhanced TRAIL-mediated apoptosis in HCC cells in the present study, HepG2 and Huh-7 cells, which are highly chemoresistant to TRAIL (17), were selected. The effect of rocaglamide on TRAIL-induced cytotoxicity was then examined using an MTT assay. Treatment with rocaglamide or TRAIL alone was minimally cytotoxic to the HepG2 and Huh-7 cells; however, pretreatment of the cells with rocaglamide enhanced the cytotoxic effect of TRAIL in a time-dependent manner in the two cell lines (Fig. 1B). Furthermore, the effect of rocaglamide on TRAIL-induced cell death was demonstrated by observing the morphological signs of apoptosis. Although rocaglamide and TRAIL alone did not induce morphological signs of cell death, rocaglamide markedly enhanced the effect of TRAIL-induced apoptosis (Fig. 1C). Cell apoptosis was also confirmed using annexin V/PI staining and flow cytometry in the HepG2 and Huh-7 cells. Treatment with rocaglamide alone led to apoptosis in ~9% HepG2 and 11% Huh-7 cells and treatment with TRAIL induced apoptosis in ~16% HepG2 and 17% Huh-7 cells. However, the combination of rocaglamide and TRAIL induced apoptosis in ~55% HepG2 and 57% Huh-7 cells (Fig. 1D and E), which is evidently more than an additive effect. A similar result was obtained by measurement of cell viability using crystal violet staining (Fig. 1F). Taken together, the data from the present study indicate that rocaglamide has the potential to sensitize highly chemoresistant HepG2 and Huh-7 cells to TRAIL-based therapy.

### Rocaglamide promotes TRAIL-induced caspase-dependent apoptotic cell death

**Figure 2:**
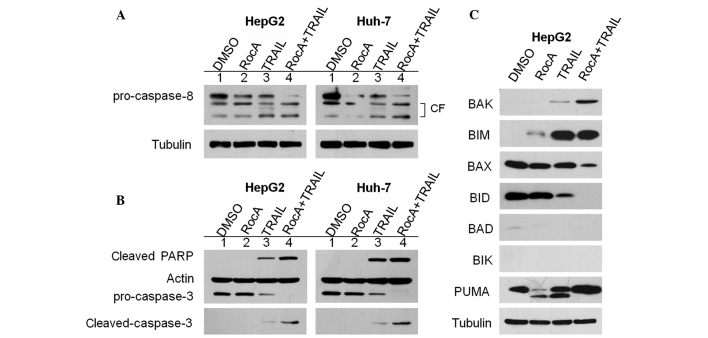
RocA enhances caspase activity triggered by TRAIL. (A and B) HepG2 and Huh-7 cells were pretreated with RocA (100 nM) for 12 h and then exposed to TRAIL (100 ng/ml) for 12 h. Cell lysates were subjected to western blot analysis using the antibodies indicated. (C) HepG2 cells were pretreated with RocA (100 nM) for 12 h and TRAIL (100 ng/ml) for 12 h. The cells were lysed and western blot analysis was performed using the antibodies indicated. The control contained cells treated with DMSO only. Results are representative of three independent experiments. RocA, rocaglamide; CF, cleaved (active) form; TRAIL, tumor necrosis factor-related apoptosis-inducing ligand; DMSO, dimethyl sulfoxide; PARP, poly ADP ribose polymerase; BAK, Bcl-2 homologous antagonist killer; BIM, Bcl-2-interacting mediator of cell death; BAX, Bcl-2-associated X protein; BID, BH3-interacting domain death agonist; BAD, Bcl-2-associated death promoter; BIK, Bcl-2-interacting killer; PUMA, p53-upregulated modulator of apoptosis.

TRAIL-induced apoptosis is mediated by activation of the caspase cascade (18). In particular, the cleavage of caspase-8 is an essential step in the TRAIL-mediated caspase activation cascade (19). Therefore, the present study investigated whether the cleavage of caspase-8 was triggered in TRAIL-resistant cell lines following treatment with rocaglamide or TRAIL alone. The results revealed that modest reductions in the level of the procaspase-8 protein occurred in the rocaglamide-treated and TRAIL-treated HepG2 and Huh-7 cells. An increase in the level of active-caspase-8 was also observed in these cells (Fig. 2A; lanes 2 and 3 vs. lane 1). However, combined treatment with rocaglamide and TRAIL significantly augmented the TRAIL-induced cleavage/activation of caspases-8 (Fig. 2A; lane 4 vs. lanes 1, 2 and 3). Notably, treatment with rocaglamide alone did not affect the expression levels of pro-caspase-3 compared with the control (Fig. 2B; lane 2 vs. lane 1). Furthermore, treatment with TRAIL alone resulted in a small reduction in the level of pro-caspase-3 and a small increase in the cleavage of poly ADP ribose polymerase (PARP) and activated caspase-3 substrates in the HepG2 and Huh-7 cells (Fig. 2B; lane 3 vs. lanes 1 and 2). However, combined treatment with rocaglamide and TRAIL resulted in significantly increased activity of the TRAIL-induced cleavage/activation of pro-caspase-3 and cleavage of PARP (Fig. 2B; lane 4 vs. lanes 1, 2 and 3), indicating that combined treatment induced apoptotic death in the hepG2 and Huh-7 cells, at least partly through a caspase-dependent pathway. Thus, these results clearly suggest that rocaglamide sensitizes TRAIL-resistant HCC cells to TRAIL-induced cell death through the enhancement of caspase activity.

As pro-apoptotic B-cell lymphoma 2 (Bcl-2) family proteins, including BH3-interacting domain death agonist (BID), Bcl-2-associated X protein (BAX) and p53-upregulated modulator of apoptosis (PUMA), are able to sensitize cancer cells to TRAIL-induced apoptosis (20), the present study investigated the expression levels of these proteins. In the HepG2 and Huh-7 cells treated with the rocaglamide/TRAIL combination, the protein levels of pro-apoptotic Bcl-2-interacting mediator of cell death (BIM), Bcl-2 homologous antagonist killer (BAK) and PUMA were significantly increased, whereas the protein levels of other pro-apoptotic proteins, including BID and BAX were reduced (Fig. 2C), indicating the possible inhibition of protein synthesis by rocaglamide (21). Therefore, these results suggest that the upregulation of the BIM, BAK and PUMA proteins is associated with the rocaglamide-mediated sensitization of HepG2 and Huh-7 cells to TRAIL-induced apoptosis. Overall, these results indicate that rocaglamide substantially increases the apoptotic potential of TRAIL in HCC cells through extrinsic and intrinsic pathways.

### Rocaglamide sensitizes TRAIL-induced apoptosis via c-FLIP downregulation

**Figure 3:**
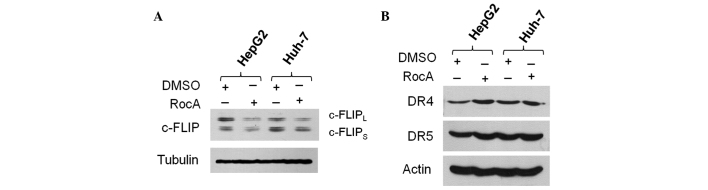
RocA downregulates c-FLIP. (A and B) HepG2 and Huh-7 cells were treated with RocA (100 nM) for 12 h. The cell lysates were analyzed using western blot analysis with the antibodies indicated. The control is represented by cells treated with DMSO only. Results are representative of three independent experiments. RocA, rocaglamide; DMSO, dimethyl sulfoxide; c-FLIP, cellular FLICE-like inhibitory protein; DR, death receptor.

c-FLIP, which is highly homologous to caspase-8 but is catalytically inactive, is able to bind to caspase-8 and the FADD and inhibit TRAIL-induced apoptosis, thus disrupting the death-inducing signaling complex (DISC) (22). Notably, HCC tumors exhibit high resistance to TRAIL due to the overexpression of c-FLIP (23). Following the observation that rocaglamide is able to overcome TRAIL-resistance in the HCC cells and activate caspase-8, the present study investigated whether this effect correlated with c-FLIP. Therefore, the effect of rocaglamide on the levels of c-FLIP, which is highly expressed in HCC cells, was examined (Fig. 3A). Notably, the resulting data also suggest that rocaglamide treatment resulted in downregulation of the c-FLIP protein in the HepG2 and Huh-7 cells (Fig. 3A). The resulting effect of rocaglamide on the levels of c-FLIP was consistent with the effects of rocaglamide on caspase-dependent apoptosis.

TRAIL triggers the extrinsic pathway of apoptosis by binding to cognate DR4 or DR5 (3), which results in the formation of receptor homotrimers and the propagation of a proapoptotic signal through caspase-8, which then self-activates and initiates downstream caspase events (24). In addition, the levels of DR4 or DR5 correlate with the sensitivity of TRAIL-mediated cell death (25). Therefore, the present study investigated the effect of rocaglamide on DR4 and DR5. No significant changes in the levels of DR4 or DR5 were observed in the rocaglamide-treated HepG2 and Huh-7 cells (Fig. 3B).

### Knockdown of c-FLIP mimics the effect of rocaglamide in overcoming TRAIL-resistance in HepG2 cells

**Figure 4:**
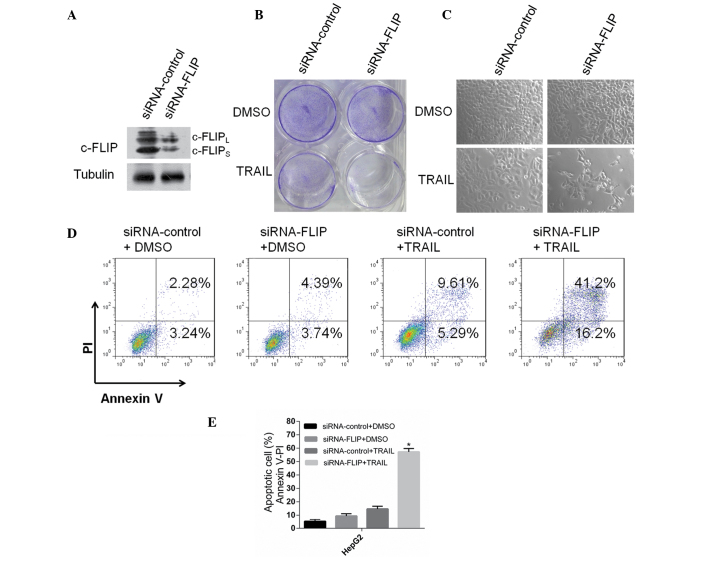
Knockdown of c-FLIP mimics the effect of rocaglamide in sensitizing HepG2 cells to TRAIL. The HepG2 cells were transiently transfected with either siRNA specific for c-FLIP or a control for 48 h and were then treated with TRAIL (100 ng/ml) for 12 h. (A) Western blot analysis indicating the expression levels of c-FLIP in the c-FLIP-silenced and control cells. (B) Cells were washed with PBS and fixed with 4% paraformaldehyde followed by staining using crystal violet. (C) Representative images of c-FLIP-silenced HepG2 and control cells. (D and E) Cell apoptosis was determined using flow cytometry with annexin/PI double staining in the c-FLIP-knockdown HepG2 and control cells (magnification, ×400). Data are expressed as the mean ± standard deviation. ^*^P<0.01, siRNA-FLIP+TRAIL group vs siRNA-control+TRAIL group. Results are representative of three independent experiments. TRAIL, tumor necrosis factor-related apoptosis-inducing ligand; DMSO, dimethyl sulfoxide; c-FLIP, cellular FLICE-like inhibitory protein; PI, propidium iodide.

In order to determine the effect of downregulation of c-FLIP on the rocaglamide-induced effect, the present study also examined whether the apoptosis-inducing effects of rocaglamide were also exerted in c-FLIP-knockdown HepG2 cells. siRNA was used to specifically downregulate c-FLIP and an siRNA vector served as a control. This treatment resulted in a marked suppression in the levels of c-FLIP of ~70% (Fig. 4A). The cell viability, evaluated by crystal violet staining, indicated that the c-FLIP-silenced HepG2 cells exhibited an increased sensitivity to TRAIL treatment compared with the that of the control (Fig 4B). In addition, the morphological signs of cell death were significantly increased following treatment with TRAIL alone in the c-FLIP-silenced HepG2 cells (Fig. 4C). Furthermore, apoptosis was analyzed using annexin V/PI staining by flow cytometry in the c-FLIP-knockdown HepG2 cells. The results revealed that the induced rate of apoptosis was ~8% by siRNA-FLIP, 15% by TRAIL and 56% by siRNA-FLIP combined with TRAIL (Fig. 4D and E), suggesting that the downregulation of c-FLIP in the HepG2 cells mimicked the effect of rocaglamide on TRAIL-mediated apoptosis in the TRAIL-resistant HCC cells.

### Rocaglamide induces apoptosis of tumor cells in SCID mice models

**Figure 5:**
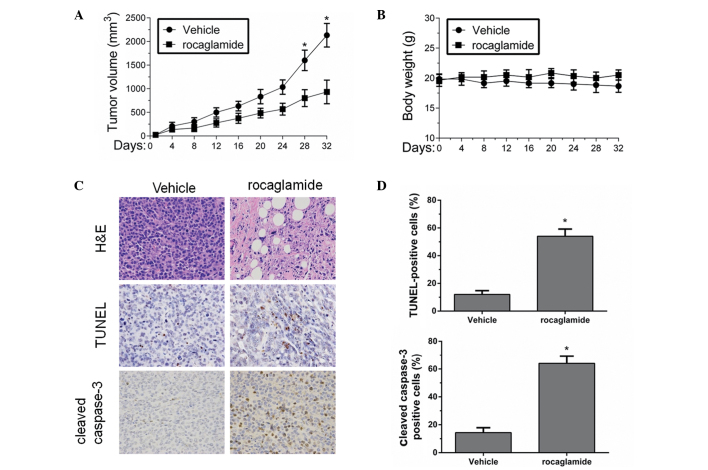
Effect of rocaglamide treatment on the growth of tumors from Huh-7 cells implanted in SCID mice. Huh-7 cell tumors established subcutaneously in SCID mice were treated with vehicle (5% DMSO in olive oil; n=5) or rocaglamide (2.5 mg/kg; n=5) via daily intraperitoneal injection into the mice for 32 days. (A) Tumor volume and (B) body weight were monitored. (C) Histological analysis of apoptosis in xenografted HCC tumors from the mice were performed using H&E, TUNEL and cleaved caspase-3 staining (magnification, ×200). (D) Quantitation of the TUNEL and cleaved caspase-3 staining in rocaglamide-treated tumors compared with vehicle-treated tumors. Data are expressed as the mean ± standard deviation. ^*^P<0.01 compared with vehicle. Data are representative of three experiments. SCID, severe combined immunodeficient; DMSO, dimethyl sulfoxide; H&E, hematoxylin and eosin; TUNEL, terminal deoxynucleotidyltransferase-mediated dUTP nick end labeling.

To confirm whether the synergistic effect of rocaglamide and TRAIL in resistant cell lines had potentially relevant clinical implications, the present study investigated the *in vivo* effect of rocaglamide and TRAIL on the growth of HCC xenograft tumors. As natural killer cells produce TRAIL *in vivo* (26,27), rocaglamide alone was administered in the *in vivo* study. The Huh-7 cells were subcutaneously injected into the right flanks of SCID mice and, when tumors were visible, the mice were matched for tumor volumes and were assigned to a control group and a rocaglamide-treated group. Tumor volumes in the rocaglamide-treated group were ~45±12% compared with the control group (Fig. 5A). Rocaglamide significantly suppressed tumor growth compared with that in the control group. Notably, treatment with rocaglamide did not lead to any reduction in body weight and no apparent signs of toxicity were observed in the mice during the treatment (Fig. 5B), suggesting that rocaglamide is generally tolerated well *in vivo*.

The present study further investigated the effect of rocaglamide on apoptosis *in vivo* by examining tumor tissues harvested from the control and rocaglamide-treated mice using H&E, TUNEL and cleaved caspase-3 staining. In the untreated controls, staining with H&E revealed a compact mass of epithelial cells, whereas following rocaglamide treatment, the tumor appearance was that of loose epithelial cell aggregates with increased interspersed mesenchymal cells (Fig. 5C, top panels). In addition, the TUNEL assays demonstrated a three-fold increase in the percentage of apoptotic cells in the rocaglamide treatment group compared with the untreated controls (Fig. 5C, middle panels and D, upper panel). Furthermore, the cleaved caspase-3 staining confirmed a five-fold increase in apoptosis in the tumor sections from the group treated with rocaglamide, relative to the untreated control (Fig. 5C, bottom panels and D, lower panel). Therefore, the *in vivo* investigation suggests that rocaglamide is an effective drug, which has the potential to inhibit the growth of HCC cell-derived tumors *in vivo*.

## Discussion

HCC is a significant cause of mortality worldwide due to its poor prognosis (28). The ability to evade apoptosis is a characteristic property of HCC cells, which results mainly from the lack of response to apoptotic stimuli (29). HCC cells have acquired drug resistance to cell death and are frequently refractory to classical chemotherapy (30). Thus, new strategies are required to address the resistance of HCC to apoptosis in order to improve the poor prognosis. Considerable attention has been directed towards investigating the effect of triggering apoptosis in HCC cells using natural products that stimulate DR-mediated apoptosis (31). In the present study, rocaglamide, a naturally occurring product, was demonstrated to sensitize TRAIL-resistant HCC cells to apoptosis through the suppression of c-FLIP in both *in vitro* and *in vivo* conditions.

As the type of cancer cell in HCC may affect the response of the cell to therapeutic agents, the robustly TRAIL-resistant HCC cell lines, HepG2 and Huh-7, were selected (16). Furthermore, the effect of rocaglamide on HepG2 and Huh-7 cells was examined, as was their sensitivity to TRAIL. Notably, the HepG2 and Huh-7 cells, were minimally responsive to treatment with TRAIL alone, indicating complete resistance to TRAIL. Treatment of the TRAIL-resistant HepG2 and Huh-7 cells with rocaglamide resulted in dose-dependent growth inhibition. Thus, these data provide evidence that rocaglamide has the potential to inhibit the proliferation of HCC cells and lead to their elimination.

TRAIL-based therapy offers promising therapeutic potential due to its specificity for cancer cells without evident adverse effects on normal cells (5,32). However, resistance to TRAIL-mediated therapy has been observed in HCC cells, which indicates that treatment with TRAIL alone may be ineffective in treating HCC. A noteworthy observation from the present study is that the HepG2 and Huh-7 cells, which are highly TRAIL-resistant, increased in sensitivity to TRAIL following pretreatment with rocaglamide, supporting the possibility of investigating rocaglamide as an adjuvant to combination therapy for HCC patients. In addition, rocaglamide triggered the process of apoptosis in the presence of TRAIL at nanomolar concentrations, suggesting the efficacy of rocaglamide may be potent and non-toxic.

The detailed mechanisms of resistance to TRAIL in HCC cells remain to be elucidated. The regulation of DISC-engaged molecules, including c-FLIP and caspase-8, has been observed to contribute to the sensitivity of TRAIL-mediated apoptosis in cancer cells (9,33,34). In addition, TRAIL resistance correlates with accelerated degradation of caspase-8 protein in cancer cells (8,35). The present study demonstrated that rocaglamide significantly activates caspase-8 in HCC cells. Furthermore, the rocaglamide-induced caspase-8 activation was found to be accompanied by increased cleavage of the apoptotic marker for caspase-3, PARP, *in vitro*. These findings are consistent with previous studies indicating that the activation of caspanse-8 induces apoptosis and sensitizes cancer cells to TRAIL (36,37).

c-FLIP inhibits the apoptotic signaling cascade by preventing the recruitment and activation of caspase-8 at the DISC (38), demonstrating that an elevated intracellular level of c-FLIP confers resistance against proapoptotic stimuli in tumor cells (39). In addition, ectopic expression of c-FLIP inhibits the release of active caspase-8 fragments from the DISC, resulting in disruption of the DISC complex (40). By contrast, downregulation of c-FLIP results in the sensitization of chemoresistant tumor cells (41). Collectively, these findings suggest that c-FLIP may be a novel therapeutic drug target for HCC. The present study provides evidence indicating that treatment with rocaglamide significantly reduces the protein expression level of c-FLIP in HepG2 and Huh-7 cells at nanomolar concentrations. Furthermore, combined treatment with rocaglamide and TRAIL significantly induced apoptosis, suggesting that rocaglamide induces sensitivity to TRAIL by the suppression of c-FLIP in HepG2 cells. Notably, siRNA was used to downregulate c-FLIP in the HepG2 cells and revealed that the downregulation of c-FLIP in HepG2 cells mimicked the effect of rocaglamide on TRAIL-mediated apoptosis in TRAIL-resistant HCC cells.

Targeting the transcriptional activation of c-FLIP is considered to be a promising approach for the downregulation of c-FLIP expression in cancer cells (42). However, agents directly inhibiting FLIP at the mRNA and protein levels remain to be elucidated. Subsequent studies are planned to determine whether rocaglamide treatment attenuates the transcriptional activation of c-FLIP are required, and to examine whether the effect of rocaglamide on c-FLIP occurs at the transcriptional level. Consistent with previous studies, the results of the present study suggest that the downregulation of the transcriptional activation of c-FLIP sensitizes HCC cells to TRAIL. However, the detailed mechanisms involved in regulating the expression of c-FLIP remain to be elucidated.

The present study demonstrated that rocaglamide, a naturally occurring product, sensitized chemoresistant HCC cells to TRAIL-mediated apoptosis by decreasing the expression of c-FLIP and activating caspase-8 *in vitro*. Furthermore, rocaglamide markedly inhibited the growth of tumors derived from Huh-7 cells *in vivo* in a xenograft mice model. Thus, these findings provide significant evidence for the development of rocaglamide as a novel therapeutic agent for use as an adjuvant to TRAIL in the treatment of HCC.
